# Modulation of double-stranded RNA pattern recognition receptor signaling in ovarian cancer cells promotes inflammatory queues

**DOI:** 10.18632/oncotarget.26378

**Published:** 2018-11-30

**Authors:** Maria Muccioli, Harika Nandigam, Tiffany Loftus, Manindra Singh, Amritha Venkatesh, Julia Wright, Michelle Pate, Kelly McCall, Fabian Benencia

**Affiliations:** ^1^ Department of Biomedical Sciences, Heritage College of Osteopathic Medicine, Ohio University, Athens, OH, 45701, USA; ^2^ Interdisciplinary Graduate Program in Molecular and Cellular Biology, Ohio University, Athens, OH, 45701, USA; ^3^ Department of Specialty Medicine, Heritage College of Osteopathic Medicine, Ohio University, Athens, OH, 45701, USA; ^4^ Diabetes Institute at Ohio University, Ohio University, Athens, OH, 45701, USA; ^5^ Biomedical Engineering Program, Russ College of Engineering & Technology, Ohio University, Athens, OH, 45701, USA; ^6^ Translational Biomedical Sciences Doctoral Program, Ohio University, Athens, OH, 45701, USA

**Keywords:** ovarian cancer, dendritic cells, dsRNA, PRR, chemokines

## Abstract

Inflammation and cancer are inter-related, and both pro- and anti-tumorigenic effects are possible in different contexts, highlighting the importance of characterizing specific inflammatory pathways in distinct tumor types. Malignant cells and non-cancerous cells such as fibroblasts, infiltrating leukocytes (i.e., dendritic cells [DC], macrophages, or lymphocytes) and endothelial cells, in combination with the extracellular matrix, constitute the tumor microenvironment (TME). In the last decades, the role of the TME in cancer progression has gained increased attention and efforts directed at abrogating its deleterious effects on anti-cancer therapies have been ongoing. In this context, we investigated the potential of mouse and human ovarian cancer cells to produce inflammatory factors in response to pathogen recognition receptor (PRR) signaling, which might help to shape the biology of the TME. We determined that mouse ovarian tumors generate chemokines that are able to interact with receptors harbored by tumor-associated DCs. We also found that dsRNA triggers significant pro-inflammatory cytokine up-regulation in both human and mouse ovarian tumor cell lines, and that several PRR can simultaneously contribute to the stimulated inflammatory response displayed by these cells. Thus, dsRNA-activated PRRs may not only constitute potentially relevant drug targets for therapies aiming to prevent inflammation associated with leukocyte recruitment, or as co-adjuvants of therapeutic treatments, but also might have a role in development of nascent tumors, for example via activation of cancer cells by microbial molecules associated to pathogens, or with those appearing in circulation due to dysbiosis.

## INTRODUCTION

Ovarian cancer is the second most common gynecologic cancer in the US and the fifth leading cause of cancer-related deaths in women. The American Cancer Society estimates that in 2017 in the United States, about 22,440 women will receive a new diagnosis of ovarian cancer and that about 14,080 women will die from ovarian cancer. Ovarian cancer can arise in germ cells, stromal cells, or epithelial cells, with over 90% of cases arising in the epithelium of the ovaries [1]. Unfortunately, ovarian cancer is typically detected late in the disease process. Not surprisingly, due to the commonality of late-stage diagnoses, nearly all cases resulting in death can be attributed to metastasis, the spreading of the tumor from the primary site to distant organs, such as the lungs or liver. Current treatments for ovarian cancer typically involve surgery and chemotherapy (taxane- or platinum-based), but considerable side effects and drug resistance are of major concern as previously reviewed [1, 2]. Thus, in addition to research directed at improving diagnostics, it is also critical to focus on novel treatment options for late-stage ovarian tumors, in particular those aiming to prevent metastasis.

The intricate relationship between cancer and the immune system has been studied extensively, and it is established that inflammation is capable of eradicating tumors, as well as promoting them, depending on the specific inflammatory context and tumor type [3–11]. Cytokines that can favor tumor regression include type-1 IFNs, IFN-γ, IL-2, IL-12, and IL-23, among others [9]. Broadly, these can activate anti-tumor immune cells, such as cytotoxic T lymphocytes (CTLs, CD8^+^ T cells) and Natural Killer (NK) cells, which can recognize danger molecules expressed on tumors and kill them directly. Often, however, cancer cells are able to evade this kind of immuno-surveillance by down-regulating these surface markers over time [12]. In fact, if the protective immune response fails to rapidly eliminate the tumor, the leukocyte populations infiltrating the neoplasm typically have a tumor-promoting inflammatory profile. High levels of tumor-associated macrophages (TAMs), CD4^+^ (helper) T cells, and T regulatory cells (Tregs) have all been implicated in favoring tumor progression [9, 13–16]. Some cytokines and chemokines secreted by these populations (and other cells in the microenvironment) that can promote tumor growth include TNF-α, IL-6, IL-8 and IL-17. Many of these cytokines are pleiotropic, having diverse effects in different inflammatory contexts and tumor types. Although the specific events by which leukocytes are recruited to the tumor site remains to be fully elucidated, locally-produced cytokines and chemokines (such as those secreted by tumor cells) may serve to attract these immune cell populations to the tumor milieu. For example, CCL5/RANTES and CCL2 are known to recruit immune cell populations like Tregs and TAMs, among others, and have been implicated in tumor aggressiveness and increased metastatic potential [17–19].

One inflammatory signaling pathway that can result in the recruitment of leukocytes begins with the activation of pattern recognition receptors (PRRs), proteins that have been extensively characterized in immune cells [20–23]. In the innate immune system, they serve to recognize specific pathogen-associated molecular patterns (PAMPs) (e.g. bacterial products like LPS or viral components such as DNA or RNA). Upon PAMP recognition, an inflammatory response results in the activation of pro-inflammatory transcription factors such as Nuclear factor kappa B (NF-ĸB), which up-regulate cytokine and chemokine production [24, 25]. Importantly, NF-ĸB has been regarded as a “master switch”, responsible for promoting tumor progression in multiple cancer types including ovarian tumors. In fact it has been shown to be constitutively activated in many cancers, and has been associated with poor clinical outcomes [26, 27]. The inflammatory cytokines and chemokines secreted as a result of NF-ĸB activation can then act to recruit immune cells to the site of inflammation via chemotaxis, for example. Thus, it has been hypothesized, that in a tumor setting, PRR activation may result in the recruitment of white blood cells to the site. PRRs are currently being explored as potential therapeutic targets for cancer treatment [28].

In addition to their expression in immune cells, such as macrophages or DCs, PRRs are also found in multiple tumor types, and thereby can act to modify the inflammatory cytokine secretion profile of the cancer cells themselves in the presence of PRR ligands [28]. With respect to ovarian cancer, tumor cell expression of high levels of Toll-like receptor 3 (TLR3), a dsRNA-sensing receptor, has been associated with tumor progression [29–31]. It is hypothesized that endogenous dsRNA (e.g. from viral infections or nearby cellular debris) can activate tumor-residing PRRs, thereby driving increased cytokine and chemokine secretion that can in turn influence the leukocyte-infiltration profile in a way that favors angiogenesis and metastasis [3, 9]. Specifically, higher levels of chemokines, such as RANTES/CCL5, may recruit more leukocytes, like TAMs and DCs capable of promoting angiogenesis at the tumor site. Indeed, the microenvironment of ovarian cancer is characterized by DCs, TAMs, and Tregs, which can secrete pro-angiogenic factors (i.e., VEGF, FGF, IL-8) and thereby drive tumor progression [14–16, 32, 33]. In fact, VEGF inhibitors are being explored as potential treatments for late-stage ovarian cancers [34].

Four known dsRNA-sensing receptors are known to date: TLR3, Melanoma-differentiation associated protein 5 (MDA5), retinoic acid-inducible gene 1 (RIG1), and protein kinase R (PKR) [35]. TLR3 resides in the endosome, whereas MDA5, RIG1, and PKR can be found in the cytosol. These PRRs are known to recognize different sizes of dsRNA, with some redundancy. It is thought that viral dsRNA, as well as dsRNA from dying cells is capable of activating these receptors, thereby triggering NF-ĸB activation. Additionally, the transcription factors AP1 and IRF3 can be activated. The PRRs use varying adaptor molecules in their signaling pathways, but converge on the activation of these transcription factors, underscoring the crosstalk between them [28, 36–38]. While AP1 activation may serve to also promote tumor growth (by increasing inflammatory cytokine levels), IRF3 activation may actually result in anti-tumorigenic effects (e.g. Type 1 interferon secretion), or might be pro-tumorigenic in tumors as well, as our previous work has shown [39]. Therefore IRF3's role in tumors is controversial as there is evidence that it could be both pro and anti-tumorigenic. Altogether, these data highlight the duality of dsRNA signaling in different tumor types and depending on the type and concentration of dsRNA activating the inflammation [30, 40].

Herewith, we identified several cytokines and chemokines produced by the microenvironment of an ovarian cancer model that match receptors harbored by tumor-associated DCs. Furthermore, we investigated the modulation of pro-inflammatory cytokine and chemokine expression by mouse and human ovarian cancer cells upon dsRNA-activated PRR signaling through polyinositic:polycytidylic acid (poly [I:C]) or polyadenylic:polyuridylic acid (poly [A:U]). Using RNA silencing technology, we were able to demonstrate that the different dsRNA-sensing PRRs expressed in mouse and human ovarian tumor cell lines collaborate in the overall inflammatory response to dsRNA stimulation. The results presented in this study will help determine whether these proteins may help shape the inflammatory component of the TME and also provide information for immunotherapeutic approaches.

## RESULTS

### Chemokines produced by the TME of an ovarian cancer model

**Figure 1:**
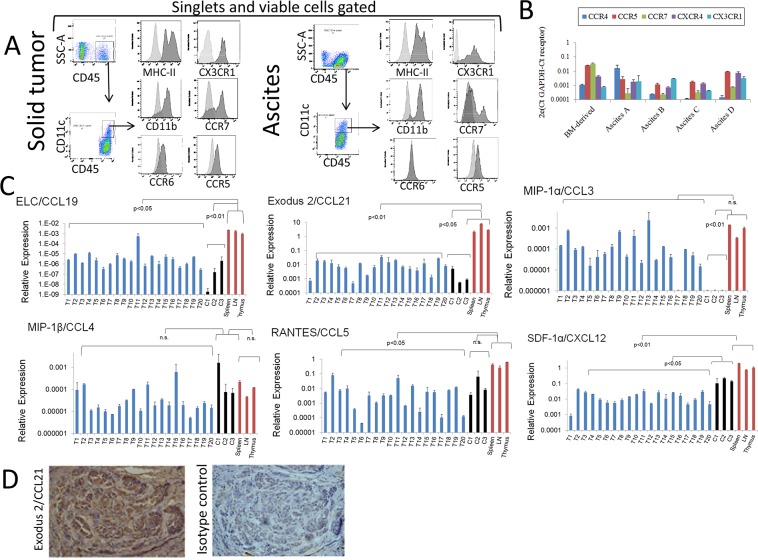
Analyses of chemokines produced by mouse ovarian tumors and expression of chemokine receptors by tumor-associated (TA)-DCs **(A)** CD45^+^CD11c^+^ cells expressing MHC-II and CD11b were detected in pooled samples of murine ovarian cancer solid tumors (mechanically disaggregated into single cell suspensions) and ascites as determined by flow cytometry. Viable cells were gated using via-Probe staining, and singlets selected by using FSC-A vs FSC-H for gating. Expression of chemokine receptors at the protein level was determined by flow cytometry analysis in these cells. **(B)** Expression of chemokine receptors at the RNA level was determined by qPCR analysis in samples from TA-DCs recovered from independent murine ovarian cancer ascites by CD11c immunomagnetic purification. RNA from bone marrow (BM)-derived DCs was also analyzed and used for qualitative comparison. These data is presented in a qualitative fashion to demonstrate the expression of those receptors in the analyzed cells. **(C)** Expression of chemokines by mouse ovarian solid tumors. Twenty ovarian ID8-VegfA tumors (T1-T20), *in vitro* cultured ID8-VegfA cancer cells (C) and normal tissues were subjected to RNA extraction followed by qPCR analysis. Data were analyzed with the Kruskal-Wallis Test (nonparametric ANOVA) followed by Dunn post-test comparisons. LN: Lymph nodes. **(D)**. Analysis of Exodus-2 at the protein level was determined in solid mouse tumor by IHC. Staining of mouse ovarian tumors with CCL21 antibody (Left Panel) and isotype control (Right Panel) shows positive staining both in tumor islets and stroma. (100X magnification).

We and others have previously demonstrated the presence of DCs in the TME of ovarian cancer and their relevance in modulating this microenvironment [41–43]. In particular, we have previously identified DC populations in the environment of mouse ovarian cancer, and were able to determine that these cells collaborate with tumor angiogenesis and progression. In order to investigate factors that might help recruit or sequester DCs by tumors, we evaluated chemokine and cytokine expression in tumor samples as well as the corresponding receptor levels in tumor-associated (TA)-DCs at the protein and RNA level. Tumors were initiated by injection of ID8-VegfA tumor cells into the peritoneal cavity or into the flanks of syngeneic and immunocompetent C57BL/6 mice, as previously described [41]. As shown in Figure 1A we detected the expression of several chemokine receptors in conventional DCs present in the microenvironment of both solid tumor and ascites by means of flow cytometry analysis. When DCs isolated from the ascites where subjected to RNA extraction and RT-PCR analysis, we obtain similar results at the RNA level (Figure 1B).

Using qPCR analysis, we analyzed chemokine expression in samples collected from 20 independent solid tumors. We compared chemokine expression to that in immune organs, as well as in cultured ID8-VegfA cells recovered from different experiments. As shown in Figure 1C, murine ovarian tumors express several chemokines at the RNA level such as ELC/CCL19 (interacts with CCR7); Exodus-2/CCL21 (interacts with CCR7); MIP-1α/CCL3 (interacts with CCR1 and CCR5); MIP-1β/CCL4 (interacts with CCR5); RANTES/CCL5 (interacts with CCR1, CCR3 and CCR5); and SDF-1α/CXCL12 (interacts with CXCR4 and CXCR7). As expected, in most cases the overall levels of chemokines produced by tumors were lower than those of immunological organs, except in the case of MIP-1α, or MIP-1β, where the expression levels were not significantly different. In addition, with respect to MIP-1α, tumor samples appear to express higher levels of the chemokine than those observed in tumor cells in culture. One possible explanation is that this chemokine is produced by tumor cells under the influence of the TME (e.g., different levels of oxygen, 3D environment, lactic acid accumulation, extracellular matrix interaction), or that other TME cells rather than cancer cells are responsible for the elevated expression of this chemokine. An immunohistochemistry analysis of solid tumors revealed the expression of Exodus 2/CCL21 at the level of protein (Figure 1D), both in tumor islets and stroma, strongly suggesting that tumor cells can be a source of chemokines *in vivo*.

Overall, these data suggest that the TME is a source of chemoattractants for immune cells; in this case, particularly DCs may be therefore attracted and sequestered by TME. Indeed, it has been previously shown that the human ovarian cancer TME is source of several cytokines and chemokines that can attract a variety of immune cells, and that this can be used advantageously when designing immunotherapeutic approaches [44].

### Murine ovarian cancer cells as a source of inflammatory factors and their modulation by double-stranded RNA-sensing pattern recognition receptors

**Figure 2:**
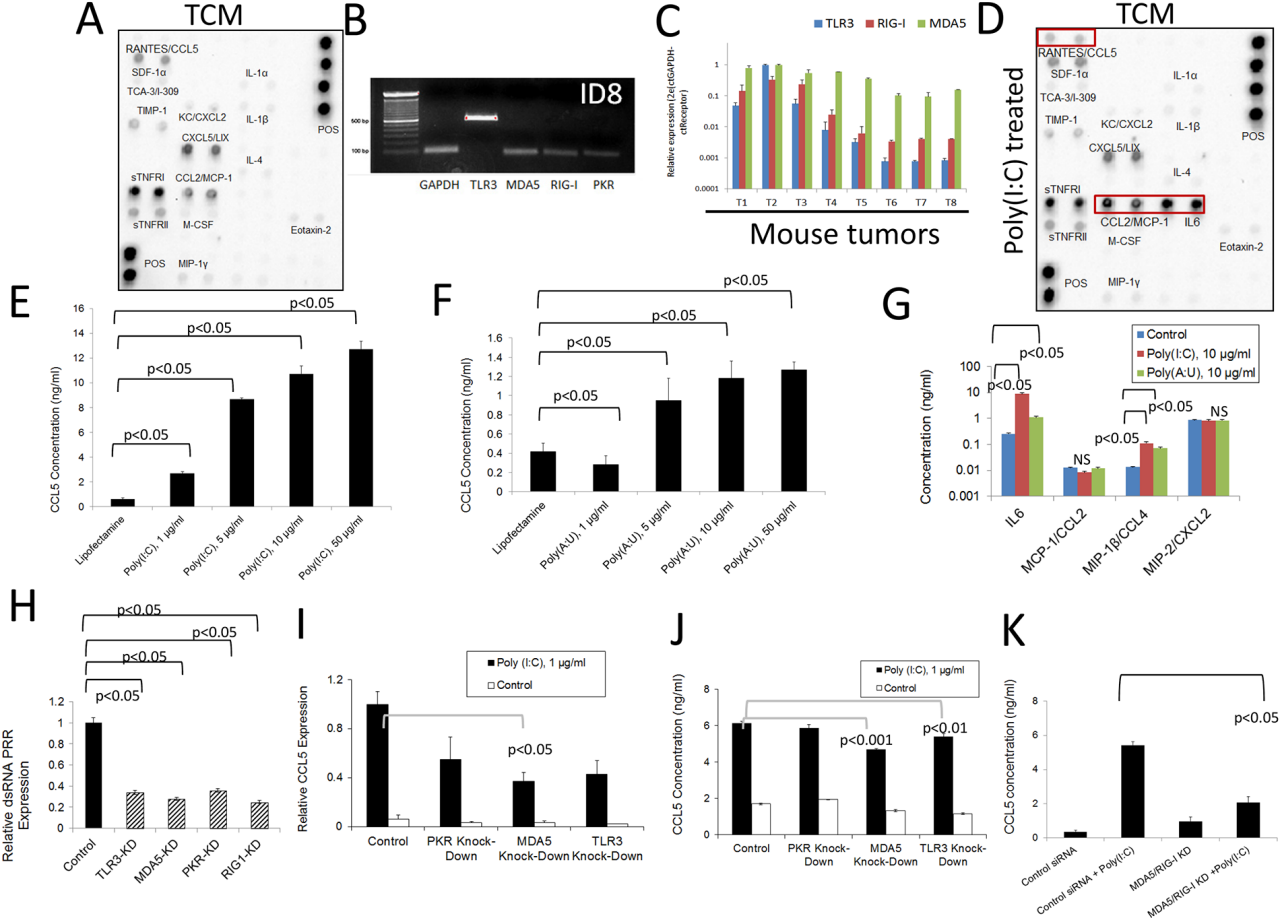
Inflammatory response of murine ovarian cancer cells to dsRNA stimulation **(A)** Antibody array analysis of inflammatory factors in supernatants of murine ovarian cancer cells mock-treated with lipofectamine. Pooled samples of independent experiments were analyzed using the RayBio® Mouse Inflammation Antibody Array 1&1.1, (RayBiotech) following the manufacturer's instructions. Pooled samples from at least three independent experiments were used for analysis. **(B)** RNA was extracted from murine ovarian cancer cells and the expression of several dsRNA PRRs was analyzed by qualitative PCR. Gels are representative of at least three independent experiments. **(C)** Expression of TLR3, MDA-5 and RIG1 was evaluated by qPCR analysis in samples from individual murine ovarian solid ID8 tumors (T1-T8). **(D)** Antibody array analysis of inflammatory factors in supernatants of murine ovarian cancer cells treated with lipofectamine plus poly (I:C). Samples were analyzed using the RayBio® Mouse Inflammation Antibody Array 1&1.1, (RayBiotech) following the manufacturer's instructions. Pooled samples from at least three independent experiments were used for the analysis. Molecules that are upregulated in the array compared to mock-treated experiments are highlighted by red boxes. **(E** and **F)** Mouse tumor cell supernatants were collected 24 hr. after transfection with synthetic dsRNA analogs, poly (I:C) and poly (A:U). ELISA was used to quantify the levels of RANTES/CCL5 in the cell supernatants. Error bars represent +/− SE. The results depicted here are representative of 3 independent experiments. **(G)** ELISA analysis of additional cytokines in the supernatants of mouse ovarian cancer cells treated as described in (E) and (F). The results presented here are representative of three independent experiments. **(H)** Expression levels of several dsRNA PRR in mouse ovarian cancer cells 24 hr. after inhibition with specific siRNAs. The results presented here are representative of at least three independent experiments. **(I** and **J)** Expression of RANTES/CCL5 at the RNA (I) and protein levels (J) in cells subjected to siRNA treatment to knock-down specific dsRNAsensing PRRs and further stimulated with poly (I:C). RNA levels were determined by qPCR and protein levels in supernatants were determined by ELISA. The results presented here are representative of at least three independent experiments. **(K)** ELISA analysis of RANTES/CCL5 in cell supernatants subjected to combined knock-down strategies and later stimulated with poly (I:C). The results presented here are representative of at least two independent experiments.

Taking into account the capability of tumors to produce DC chemoattractants as described above, we decided to determine the capacity of tumor cells, major (but not unique) components of the TME, to produce chemotactic factors. Analysis of tumor conditioned media (TCM) at the protein level using antibody arrays (Figure 2A) showed complementary results to those presented in Figure 1C and 1D. Densitometry analysis comparing the molecules expressed at higher levels upon treatment is presented in Supplementary Figure 1A. These data indicate that tumor cells produce several inflammatory factors at the protein level that may help recruit immune cells to the TME, as well as affect their function upon arrival. In order to determine which pathways may modulate the expression of these factors by tumor cells, we investigated the expression of PRRs in mouse ovarian cancer cells. As shown in Figure 2B, these cancer cells express PRRs which are able to respond to double-stranded (ds)RNA such as TLR-3, RIG1, MDA5 and PKR. The expression of these receptors has been previously demonstrated in human ovarian cancer cells as we have reviewed [45, 46]. In addition, we determined that these receptors are expressed in independent samples of solid tumor tissues at the RNA level (Figure 2C and Supplementary Figure 1B). As expected, the expression of these receptors was higher in samples from immune organs compared to other tissues in healthy mice (Supplementary Figure 1C). We also determined that murine ovarian cancer cells express other PRRs at the RNA level, including TLR2, TLR4 and TLR7, indicating that several innate immune inflammatory pathways can be activated in these cells (Supplementary Figure 1D) in response to microbial molecules from microbial pathogens, microbes the oncobiome or microbial products in circulation as a result of intestinal dysbiosis.

We next investigated the effect of dsRNA signaling on these ovarian cancer cells by using synthetic dsRNA (poly [I:C] or poly [A:U]) transfection. To characterize the cytokine and chemokine expression profile of murine ovarian cancer cells in response to this treatment, we used a combination of qPCR, ELISA, and antibody array experiments. Synthetic dsRNA is known to have an effect on cells (with respect to the activation of inflammatory signaling pathways and increased cytokine and chemokine secretion) at a broad concentration range (i.e. 0.1-100 μg/ml). In fact, high molecular weight (HMW) poly (I:C) has been used for many decades to stimulate TLR3 in multiple cells lines and is a well-established activator of TLR3 signaling [47]. At higher concentrations (i.e. 50-100 μg/ml for poly [I:C]), dsRNA transfection has been shown to trigger apoptosis in some responsive cell lines [37]. We hypothesize that such high dsRNA concentrations are unlikely to be present in the TME, and that lower concentrations (i.e. 1-10 μg/ml) are likely to be more relevant when studying the effects of these signaling pathways on tumor progression. As depicted in Figure 2D, several inflammatory molecules, including RANTES/CCL5, MCP-1/CCL2, and IL-6 were upregulated after transfection of murine ovarian tumor cells O/N with poly (I:C) (10 μg/ml), compared to the untreated cell supernatants (Figure 2A). RANTES/CCL5 is a pluripotent chemokine that can attract a variety of T cell populations (e.g. Tregs and effector memory T cells (CD4^+^/CD45RO^+^) as well as eosinophils, basophils, monocytes, B cells, NK cells, and immature DCs [9]. IL-6 is a known inducer of tumor-promoting inflammation that can activate a variety of proliferative cellular pathways including MAPKs, PI3Ks, and STATs, among others [48–50]. Furthermore, MCP-1/CCL2 can recruit several leukocyte subpopulations, including monocytes and DCs, which can stimulate angiogenesis by secreting factors such as TNF-α [9, 17, 19].

Next, we focused on determining RANTES/CCL5 expression in ovarian cancer in response to dsRNA. A dose-response analysis of the capacity of dsRNA to induce RANTES/CCL5 expression is presented in Figure 2E-2F. As can be seen, this molecule was significantly upregulated at the protein level following transfection with HMW poly (I:C) (which can activate TLR3, MDA5, and PKR) and also after transfection with poly (A:U) (a synthetic ligand that predominantly activates TLR3) [51]. Poly (A:U) transfection produced a less pronounced increase in cytokine expression, suggesting that while TLR3 is certainly involved in dsRNA-induced signaling that results in increased cytokine production, other receptors (such as MDA5 or PKR) are also part of this process in this mouse epithelial ovarian tumor cell line. We found that statistically significant cell death occurs with poly (I:C) transfection at concentrations higher than 10 μg/ml and that of poly (A:U) at 5μg/ml and above as determined by dose-response *in vitro* viability studies (Supplementary Figure 1E-1F). Additionally, we validated the protein array data with respect to IL-6 expression by means of ELISA experiments (Figure 2G). On the contrary, no differences in MCP-1/CCL2 expression were observed when using this technique. We also found that MIP-1β/CCL4 is upregulated upon transfection with both poly (I:C) and poly (A:U). CXCL2, was present in the supernatants of mouse ovarian tumor cells (Figure 2A), but not upregulated upon dsRNA transfection as determined by array analysis (Figure 2D), and also showed no differences when analyzed by ELISA. Thus, both RANTES/CCL5 and IL-6 are molecules that were upregulated upon dsRNA transfection of cancer cells at the protein level as determined by two complementary methods. It has been reported that dsRNA can promote the upregulation of dsRNA-sensing PRRs in some cells [52]. In our studies we were able to determine, at the level of RNA, that PKR was the only dsRNA PRR affected by the transfection in these murine ovarian cancer cells, and only upon transfection with poly (A:U), indicating that PKR may participate in a positive feedback loop in response to dsRNA stimulation (Supplementary Figure 1G).

PRR polymorphisms have been implicated in poor clinical outcomes in some cancers, an example of which is the overexpression of TLR3 in human ovarian tumors [29]. To further understand the mechanisms by which dsRNA signaling can promote chemokine expression in tumor cells, we set out to determine the involvement of the different dsRNA-sensing receptors (and thus the downstream inflammation) in these tumor cells. To this end, we employed siRNA technology to target three of the four receptors (TLR3, MDA5, and PKR) and knock-down the expression of these molecules. HMW poly (I:C) was then used to treat the cells, and the RANTES/CCL5 levels were quantified in order to determine the contribution of each receptor to dsRNA-induced inflammation in this model. The efficacy of the knockdown strategy was evaluated by qPCR. An approximate 70-80% reduction in the RNA levels was achieved with the targeting siRNA compared to the control siRNA for each gene of interest (Figure 2H). We observed that this treatment induced a decrease in the expression of TLR3 at the protein level (Supplementary Figure 1H). As shown in Figure 2I and 2J, siRNA inhibition decreased the expression of RANTES/CCL5 in response to poly (I:C) at the level of both RNA and protein. RANTES/CCL5 was chosen to be the “read-out” of PRR activation and downstream signaling, since it was significantly and consistently upregulated after HMW poly (I:C) transfection, even at low concentrations (Figure 2E). None of the independent knock-down treatments were able to completely abolish the induction of RANTES/CCL5, suggesting that several receptors are responding simultaneously to the dsRNA stimulation. The strongest inhibitory response was observed when a double knock-down strategy was employed (siRNA targeting MDA5 and RIG1). Although RIG1 primarily responds to low molecular weight (LMW) dsRNA, its downregulation in combination with MDA5 knockdown further diminished the effect observed when knocking down MDA5 alone (Figure 2K). Finally, taking into account that siRNA can form structures that may be able to interact with dsRNA PRRs, we evaluated if the siRNA inhibitors by themselves were able to induce an inflammatory response in our cells. Interestingly, we did not observe any upregulation in the expression of RANTES/CCL5 using siRNA transfection alone (Supplementary Figure 1I-1J). Altogether, these data demonstrate that several dsRNA receptors simultaneously participate in sensing dsRNA in murine ovarian cancer cells. This suggests that several molecules may have to be targeted in ovarian cancer cells to effectively decrease inflammatory signals enabled by dsRNA in this model. On the other hand, targeting one molecule may be enough if it is the one mainly responsible for the dsRNA inflammatory response in a particular cell line, or in a patient's tumor.

### Human ovarian cancer cells as source of inflammatory factors and their modulation by dsRNA-sensing PRRs

**Figure 3:**
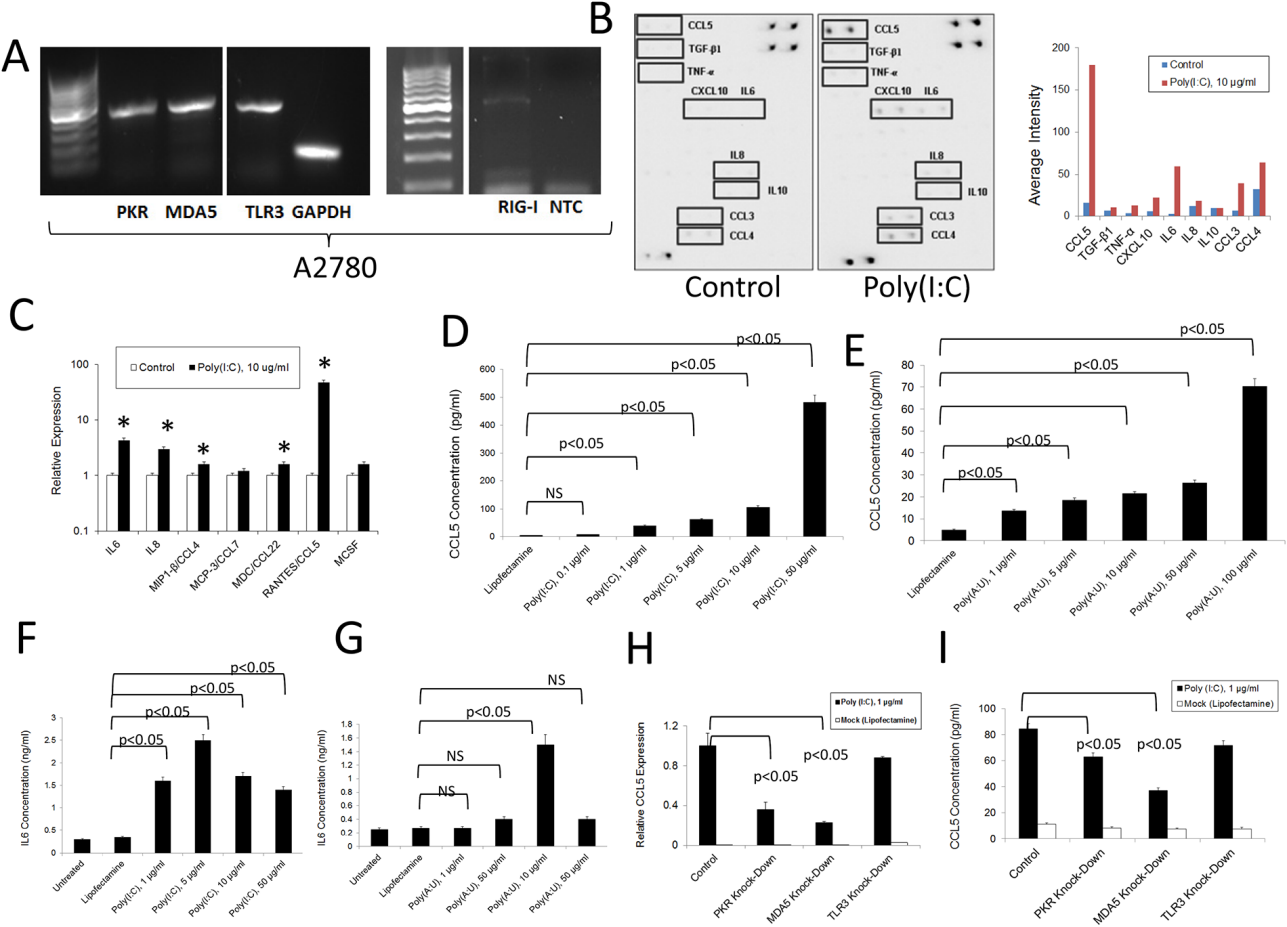
Inflammatory response of human A2780 ovarian cancer cells to dsRNA stimulation **(A)** RNA was extracted from A2780 ovarian cancer cells and the expression of several dsRNA PRRs was analyzed by qualitative PCR. The gels are representative of at least three independent experiments. NTC: Non-template control. NTC were also negative for PKR, MDA5 and TLR3 (as shown in Supplementary Figure 3) **(B)** Antibody array analysis of inflammatory factors in supernatants of human A2780 ovarian cancer cells mocktreated with lipofectamine or stimulated with poly (I:C). Pooled samples of independent experiments were analyzed using the RayBio® Human Inflammation Antibody Array following the manufacturer's instructions. Pooled samples from at least three independent studies were used for the experiment. Densitometry analysis was performed by using the ImageJ software and data represented as bars for comparison. **(C)** QPCR analysis of the RNA expression of several chemokines/cytokines in human ovarian cancer cells treated as described in (B). Data is representative of at least three independent studies. ^*^ p<0.05, when lipofectamine-treated (mock) samples were compared to poly (I:C) treated samples for the same chemokine/cytokine. **(D** and **E)**. Tumor cell supernatants were collected 24 hr. after treatment with synthetic dsRNA analogs, poly (I:C) and poly (A:U). ELISA was used to quantify the levels of RANTES/CCL5 in the cell supernatants. Error bars represent +/− SE. The results depicted here are representative of three independent experiments. **(F** and **G)**. Tumor cell supernatants were collected 24 hr. after treatment with synthetic dsRNA analogs, poly (I:C) and poly (A:U). ELISA was used to quantify the levels of IL-6 in the cell supernatants. Error bars represent +/− SE. The results depicted here are representative of three independent experiments. **(H)** and **(I)**. Expression of RANTES/CCL5 at the level of RNA (H) and protein (I) in cells subjected to siRNA transfection to knockdown specific dsRNA-sensing PRRs and further stimulated with poly (I:C). RNA levels were determined by qPCR and the protein levels in the supernatants were determined by ELISA. The results presented here are representative of at least three independent experiments.

To determine the relevance of dsRNA signaling in human ovarian cancer cells, we first investigated the effect of dsRNA-sensing PRR activation on the widely-used A2780 human ovarian cancer cell line. As shown in Figure 3A, as was the case in the murine system, these cells express several dsRNA PRRs as determined by qualitative PCR analysis. Upon O/N treatment with poly (I:C), these cells upregulated expression of RANTES/CCL5, IP10/CXCL10, IL-6, CCL3, CCL4 and IL-8, but not the anti-inflammatory molecules IL-10 or TGF-β1, as determined by antibody array experiments (Figure 3B). Analysis of the same molecules at the level of RNA showed a significant increase in the expression of IL-6, IL-8, CCL4, and CCL22 in cells treated with poly (I:C) (Figure 3C). As was the case in the murine cells, a dose-dependent effect was observed in the expression of RANTES/CCL5 at the protein level following transfection with poly (I:C) or poly (A:U) (Figure 3D and 3E). Similarly, as shown in Figure 3F and 3G, IL-6 protein expression by A2780 cells increased in response to dsRNA transfection, although expression decreased at higher concentrations, probably due to toxicity associated with treatment at high concentrations (Supplementary Figure 2A). Interestingly, dsRNA transfection upregulated the expression of MDA5, RIG1 and PKR, suggesting a broad positive feedback mechanism in this human ovarian cancer cell line (Supplementary Figure 2B). Finally, as with murine ovarian cancer cells, knocking down individual dsRNA PRRs did not completely abolish the induction of RANTES/CCL5 at the level of RNA or protein upon stimulation with dsRNA, suggesting a contribution of several receptors to the inflammatory response studied here (Figure 3H and 3I). We also found a significant reduction in cell viability after transfection with poly (I:C) but not poly(A:U) at the concentration of 10 μg/ml and above (Supplementary Figure 2A). In the case of poly (A:U), a significant decrease in cell viability was observed with concentrations at and above 50 μg/ml. In addition, poly (I:C) transfection induced upregulation of MDA5 and PKR, indicating a positive feedback mechanism. Interestingly, poly (A:U) transfection was only able to increase RIG1 expression. (Supplementary Figure 2B).

**Figure 4:**
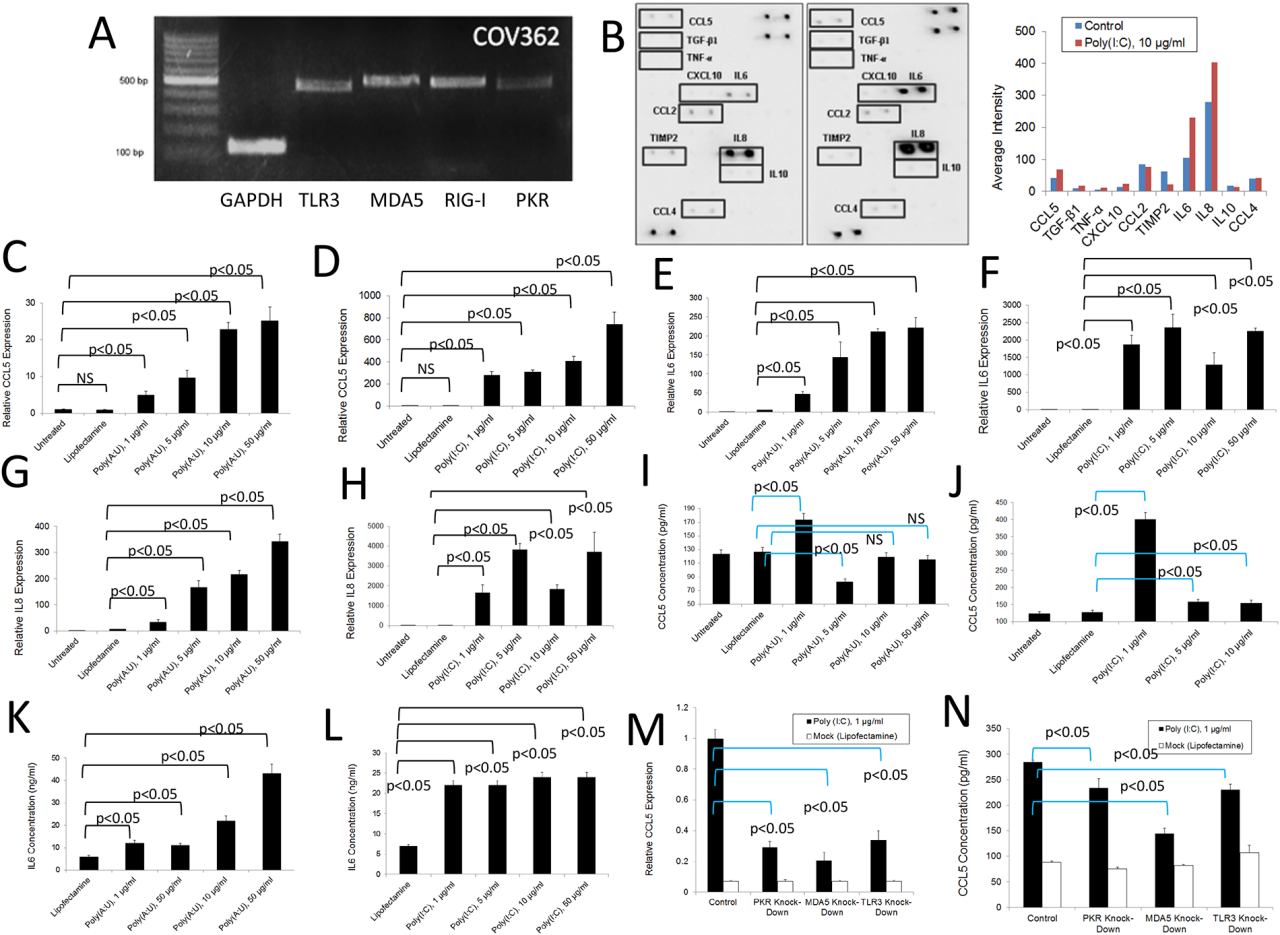
Inflammatory response of human COV362 ovarian cancer cells to dsRNA PRR stimulation **(A)** RNA was extracted from COV362 ovarian cancer cells and the expression of several dsRNA PRRs analyzed by qualitative PCR. Gels are representative of at least three independent studies. **(B)** Antibody array analysis of inflammatory factors in supernatants of human COV362 ovarian cancer cells mock-treated with lipofectamine or stimulated with poly (I:C). Pooled samples of independent experiments were analyzed using the RayBio® Human Inflammation Antibody Array following the manufacturer's instructions. Pooled samples from at least three independent experiments were used for the analysis. Densitometry analysis was performed by using the ImageJ software and data represented as bars for comparison. **(C** and **D)** QPCR analysis of the RNA expression of RANTES/CCL5 in COV362 human ovarian cancer cells treated as with poly (I:C) or poly (A:U). Data is representative of at least three independent experiments. **(E** and **F)** QPCR analysis of the RNA expression of IL-6 in COV362 human ovarian cancer cells treated with poly (I:C) or poly(A:U). Data is representative of at least three independent experiments. **(G** and **H)** QPCR analysis of the RNA expression of IL-8 in COV362 human ovarian cancer cells treated with poly (I:C) or poly (A:U). Data is representative of at least three independent experiments. **(I** and **J)** ELISA analysis of the expression of RANTES/CCL5 in supernatants of COV362 human ovarian cancer cells treated as with poly (I:C) or poly (A:U). Data is representative of at least three independent studies. **(K** and **L)** ELISA analysis of IL-6 expression in supernatants of COV362 human ovarian cancer cells treated with poly (I:C) or poly(A:U). Data is representative of at least three independent experiments. **(M** and **N)** Expression of RANTES/CCL5 at the level of RNA (M) and protein (N) in COV362 cells subjected to siRNA transfection to knock-down specific dsRNA-sensing PRRs and further stimulated with poly (I:C). RNA expression levels were determined by qPCR and the protein levels in the supernatants were determined by ELISA. The results presented are representative of at least three independent experiments.

Taking into account studies that indicate that the A2780 cell line (histology: adeno-carcinoma, characterization: tumor tissue) is highly differentiated due to its history of culture, we decided to include the ovarian cancer COV362 cell line (histology: endometrioid; characterization: pleural effusion) in our studies because it reportedly behaves similarly to primary cancer cells, being likely high-grade serous [53]. As shown in Figure 4A, COV362cells express various dsRNA-sensing PRRs as determined by qualitative PCR analysis. We determined that upon stimulation with poly (I:C), expression of some cytokines and chemokines at the protein level (RANTES/CCL5; IL-6, and IL-8 notably) is increased as determined by antibody array experiments (Figure 4B). To study this phenomenon in more detail, we first analyzed the dose-dependent responses of COV362 cells to poly (A:U) and poly (I:C) (Figures 4C-4H) by measuring RANTES/CCL5, IL-6 and IL-8 expression at the level of RNA. As shown in Figure 4C, 4E and 4G, poly(A;U) transfection, designed to predominantly stimulate TLR3, induced - typical dose-response curves with respect to the expression of the studied molecules at the RNA level. Poly (I:C) transfection, which simultaneously targets several dsRNA receptors, also induced a typical dose-response effect on RANTES/CCL5 RNA expression (Figure 4D). However, in the case of IL-6 and IL-8, a slight decrease in the expression of both molecules was observed at the transfection concentration of 10 μg/ml compared to 1 or 5 μg/ml transfection (Figure 4F and 4H). Nevertheless, we observed a significant increase in the expression of these molecules with respect to the control treatment for all of the assayed poly (I:C) concentrations. With respect to protein levels, we observed similar responses as those observed at the RNA level, both for poly (I:C) and poly (A:U) transfections (Figure 4I and 4J). The poly (I:C) data is consistent to what we observed in the cytokine arrays. On the other hand, a robust increase in IL-6 protein levels was observed both with poly (I:C) and poly (A:U) stimulation (Figure 4K and 4L). We observed a significant reduction in cell viability when treating the cells with poly (I:C) and poly (A:U) at a concentration of 10 μg/ml and higher (Supplementary Figure 2C). Furthermore, poly (I:C) transfection induced upregulation of TLR3 and PKR, again suggesting a positive feedback mechanism induced by dsRNA signaling (Supplementary Figure 2D). Similar to what was found in the murine cell line (ID8-VegfA) and the human cell line A2780, knocking down individual dsRNA PRRs did not entirely abolish the inflammatory response of these cells, once again highlighting the participation of various receptors in the overall observed effect (Figure 4M and 4N). Importantly, as is the case with the murine studies, the siRNA reagents used here were not able to induce by themselves an activation of these human cancer cell lines studied (data not shown). Finally, we were able to observe similar inflammatory responses upon stimulation of another human ovarian cancer cell line (SKOV3) with dsRNA, as determined by qualitative PCR analysis, antibody array analysis and qPCR studies (Supplementary Figure 3A-3C).

## DISCUSSION

It is well-established that the relationship between the immune system and cancer plays a significant role in tumor progression or regression, and that a combination of processes occurring at the tumor site dictate the particular outcome. The specific population of leukocytes infiltrating the tumor comprises a critical component in determining disease outcome, highlighting the importance of characterizing processes leading up to leukocyte recruitment. Of the characterized processes leading up to local inflammation that attracts immune cells, the activation of innate immune receptors such as PRRs may constitute a major starting point of inflammation. This can result in the recruitment of tumor-promoting white blood cells in several cancer types, particularly in primary tumors that have not yet gone through the angiogenic switch, or in secondary tumors that can start growing in the context of metastasis. In this regard, although great advances have been made in our knowledge of the TME in the last decade, the interplay between PRR activation in immune cells and tumor cells remains to be fully elucidated.

Here, we focused on the dsRNA-activated pathways in mouse and human ovarian cancer cells. The results presented here support further investigation on the role that PRR activation in tumor cells may have in contributing to the overall inflammatory profile at the tumor site. We are particularly interested in the role of DCs, since these cells are keystones in the development of specific immune responses, and a deviation from their normal functions, either by reprogramming or by their sequestration in the TME, can greatly hamper the ability of the immune system to mount a specific antitumor response. As shown in Figure 1, the TME of this ovarian cancer model harbors DCs, in line with what we and others have previously shown both in this mouse model and human ovarian cancer [41, 42]. Here, we have demonstrated that the TME produces chemoattractants that match receptors on the surface of tumor-associated DCs. These data suggest that DCs can be recruited to tumor sites from lymphoid organs, and also that the TME has the capability to retain those cells in its midst. This is relevant, because once DCs are loaded with antigen in the periphery, in this case in the TME, they need to return to a lymphatic organ in order to activate T cells. Therefore, hampering DC migration will impair the development of a robust immune response against cancer as previously reported as a mechanism of immunoescape [54]. In addition, factors from the TME, such as VEGF, can prevent DC maturation, thus rendering them incapable of activating T cells or inducing T cell anergy [55]. As we and others have shown, DCs may also contribute to neoangiogenesis in pathological conditions via unloading of angiogenic factors at the tumor site, or by directly participating in the development of neovessels [43]. This knowledge can help engineer DC based vaccines for therapeutic vaccination that are impervious to these chemoattractants, or on the other hand, take advantage of those chemoattractants to deliver DC vaccines, engineered to be impervious to the TME suppressive factors with the aim of inducing intratumor vaccination. Indeed, taking advantage of ovarian cancer TME chemokines for adoptive therapy approaches has been recently described [44].

In the present study we demonstrated that an array of chemotactic factors and/or inflammatory factors is generated in the TME of murine ovarian cancer. As our study shows, some of these factors can be produced directly by tumor cells (with cells of the stroma in a solid tumor, or non-transformed cells from the ascites also likely contributing to the inflammatory cytokine/chemokine milieu). A growing body of evidence highlights the capability of tumor cells to generate inflammatory factors, and thus shape its environment (reviewed in [56]). As shown here, both murine and human ovarian cancer cells express several dsRNA-sensing receptors, and all of them appear to contribute to some extent to the inflammatory response induced by dsRNA stimulation. This is in line with previous studies in ovarian cancer cells, and other cancer cell types demonstrating expression of PRRs by these cells (reviewed in [46]). The use of dsRNA in a therapeutic setting, in particular poly (I:C) has been largely explored. It is well-known that high doses of poly (I:C) can induce tumor cell death, but clinical efforts were hampered in the past due to its toxicity [57]. In recent years, antitumor therapeutic approaches have focused on inducing immunogenic cell death (e.g. inducing the death of tumor cells while attracting immune cells that are capable of phagocytosis and are activated in the process). In fact, PRRs are considered to be target molecules for *in situ* vaccination [58]. Our results contribute novel and relevant information to the ongoing pursuits of targeting dsRNA PRRs for cancer. Since different PRRs respond to dsRNA stimulation, strategies to target them individually, therefore narrowing the extent of the inflammatory response induced, are promising. This takes into account that inflammation is a double-edged sword in the context of cancer, which may contribute to tumor development or tumor regression or prevention (the latter as postulated by the tumor immune surveillance theory). In addition, strategies to activate inflammation only in the antigen presenting cell population (i.e., DCs or macrophages), or the tumor cells may be useful. Another important point is that we observed a variation in the susceptibility to dsRNA transfection among different cell lines, or between ovarian cancer cells from different origin and histology. In this case, A2780 and COV362 responded differently with regard to toxicity and upregulation of PRRs induced by dsRNA transfection. Therefore, it will be important to analyze the patient's own tumor cell susceptibility to the treatment in order to determine the most favorable course of treatment (for example, overall inflammation leading to neoangiogenesis vs. activation of APCs) (i.e., personalized medicine). In addition, the data presented here will contribute to a better understanding of the phenomena of immunogenic cell death and *in situ* vaccination. As shown here, even when cells are killed by high concentrations of dsRNA-sensing PRR agonists, they are able to produce factors that attract immune cells. This might be relevant not only for therapies using dsRNA constructs, but can also help to interpret the efficacy of oncolytic virotherapy. We have previously demonstrated that virus-induced death of cancer cells can turn them into effective immunogens [59] and that oncolytic virotherapy in the same ovarian cancer model used in the current studies not only induces *in situ* expression of chemoattractants but also determines an increase of activated TME DCs harboring tumor antigen [60]. As described above, viral infections are the main activators of dsRNA-sensing PRRs, thus activation of these pathways may contribute to the efficacy of the oncolytic virotherapy.

Finally, it is tempting to envision a role of infections on the activation of inflammatory signals by cancer cells in nascent tumors (or leftover tumor tissue upon debulking in ovarian cancer), thereby promoting the angiogenic switch via attraction of inflammatory cells to their midst. It has been recently demonstrated that ovarian cancer has a particular microbiome (oncobiome) that harbors distinct viral, bacterial, fungal and parasitic signatures [61]. Of particular interest for dsRNA-sensing PRR signaling, signatures from both positive and negative sense single-stranded RNA virus were detected. Negative-stranded RNA viruses typically generate dsRNA structures during their infection and/or replication cycles, thus being able to promote activation of the receptors studied herewith. In addition, bacterial signatures were also detected as part of the ovarian cancer oncobiome. Altogether, this indicates that ovarian cancer cells, which harbor not only dsRNA-sensing PRRs, but other PRRs which can interact with bacterial compounds, can be influenced by the oncobiome. Finally, the presence of functional PRRs in ovarian cancer cells should be considered when designing therapies that are vector-based (e.g., oncolytic viruses or bacteria-based vaccines) or when using PAMPs to stimulate the innate immune response or to increase vaccination efforts in the context of ovarian cancer therapy.

## MATERIALS AND METHODS

### Animals

Six to eight week old female C57BL/6 mice (H-2Kb, Charles River Laboratories, Wilmington, MA) were used as a source of bone marrow precursors for the *in vitro* differentiation of DCs or for the induction of ectopic and orthotopic ovarian tumors. All the animal experiments described in these studies were conducted under protocols approved by the Ohio University Institutional Animal Care and Use Committee.

### Cell lines and tumors

Murine ID8-VegfA [62] and human A2780 and COV362 (ATCC, Manassas, VA) epithelial ovarian carcinoma cells were cultured in Dulbecco's Modified Eagle's Medium (DMEM; Gibco, Grand Island, NY) with 10% fetal bovine serum (FBS) and an antibiotic cocktail (antibiotic/anti-mycotic, Gibco, Grand Island, NY) at 37°C and 5% CO_2._ The ID8 cell line is a tumor cell line derived from spontaneous *in vitro* malignant transformation of C57BL/6 mouse ovarian surface epithelial cells originally generated by Roby *et al.*, [63]. This line has been engineered to express high levels of VEGF-A (VEGF-164) as we previously described [62].

Ectopic ID8-VegfA solid ovarian tumors were established by subcutaneous (s.c) injection of 7×10^6^ cancer cells in the flank of C57BL/6 mice [60, 62, 64]. Intraperitoneal tumors were initiated in C57BL/6 mice by intraperitoneal (i.p.) injection of 5×10^6^ cancer cells [60]. In the intraperitoneal model, animals develop ascites in approximately 30 days.

In some experiments, we prepared single-cell suspensions from solid tumors and ascites. To achieve this, solid tumors were excised, minced with scissors in cold PBS and filtered through 70 μm cell strainers (BD Biosciences, San Jose, CA) as we have previously described [65]. With respect to ascites, the cellular fraction was obtained upon centrifugation of fluid recovered from mice harboring ID8-VegfA orthotopic tumors. Upon centrifugation, both supernatants and cellular fractions were used in the studies (the latter after red blood cell lysis).

### *In vitro* generation of murine myeloid DCs

Mouse DCs were differentiated from bone marrow precursors upon culture with granulocyte-macrophage colony-stimulating factor (GM-CSF) as previously described [66–69]. The bone marrow cells recovered from flushing femurs and tibiae of C57BL/6 mice were cultured in RPMI-1640 supplemented with antibiotics, L-glutamine (2 mM) and 10% FBS (all Invitrogen, Carlsbad, CA) in the presence of 20 ng/ml of murine GM-CSF (315-03, PeproTech Inc., Rocky Hill, NJ) for 8 days. Medium containing GM-CSF was replenished on days 3 and 6.

### Immunomagnetic purification of CD11c cells from ascites

CD11c cells were isolated from ascites by magnetic sorting. To this end, ascites were collected from tumor bearing mice and then red blood cells were eliminated by hypotonic shock. After removing dead cells (Dead Cell Removal kit, MACS Miltenyi, Auburn, CA), the Fc receptors were blocked (Fc block, 2.4G2; BD Biosciences, San Jose, CA), and the cells were labeled with anti-CD11c magnetic beads (MACS Miltenyi, Auburn, CA). Next, CD11c-positive cells were isolated by using an octoMACS magnet harboring MS paramagnetic columns (all MACS Miltenyi). To increase purity, labeled cells were subjected to two successive purifications.

### Flow cytometry analysis

A FACSAria II SORP flow cytometer (Becton Dickinson, San Jose, CA) was used for acquisition and the data was analyzed with FlowJo software (FLOWJO, LLC, Ashland, OR). Non-specific binding was prevented by treating cells with Fc block (BD Biosciences, San Jose, CA) in FACS buffer (2% FBS, 0.05% sodium azide in PBS) prior to staining. Live cells were determined using Via-Probe (BD Biosciences) staining following the manufacturer's instructions. The following antibodies were used in our studies: CD11c (HL3), CD11b (M1/70), CD45 (30-F11), CCR5 (7A4), CCR7 (4B12), (all eBioscience, San Diego, CA); CCR6 (FAB590F) R&D Systems (Minneapolis, MN), MHC-II (AF6-120.1), (BD Biosciences); and CX3CR1 (sc20432) Santa Cruz Biotechnology (Santa Cruz, CA).

### Synthetic dsRNA transfection

HMW poly (I:C), poly (A:U), and lipofectamine (transfection reagent) were purchased from Invivogen (San Diego, CA) and used in accordance with the manufacturer's instructions. Concentrations of lipofectamine were adjusted depending on the concentration of poly (I:C) (concentration range: 1-50 μg/ml). Cells were grown in DMEM with 10% FBS and antibiotics (Anti-Anti; Gibco, Grand Island, NY) and allowed to attain 75-90% confluence before transfection. The media was changed to OptiMEM (Gibco, Grand Island, NY) directly prior to the dsRNA transfection. All samples were incubated with poly (I:C) admixed with lipofectamine or lipofectamine only (mock treatment) for 24 hr., after which cells and supernatants were collected for analysis.

### Small inhibitor (si)RNA transfections

siRNA targeting human and mouse TLR3, MDA5, RIG1, and PKR and a control siRNA sequence not expected to target any human or mouse genes were purchased from ThermoScientific (Waltham, MA). The siRNA transfections were carried out according to the manufacturer's instructions, with lipofectamine (Invivogen, San Diego, CA) as the transfection reagent, using the lowest possible concentration of siRNA and lipofectamine effective for knock-down. Cells were incubated for 24 hr. after which they were either collected for analysis or subjected to dsRNA transfection (above). All knock-downs were verified at the RNA level using real time quantitative PCR-(qPCR) following total RNA extraction and reverse-transcription.

### RNA extraction

The Qiagen Mini RNA Extraction kit (Valencia, CA) or Promega RNA extraction kit (Madison, WI) were employed for total RNA extraction from mouse and human cells, following the instructions provided by the manufacturer. The RNA was subjected to DNAse digestion, and cDNA was prepared using the High Capacity Reverse Transcription kit from Applied Biosystems (Grand Island, NY).

### RT-PCR

RT-PCR was used to confirm the expression of TLR3, MDA5, RIG1, and PKR in the ID8-VegfA mouse ovarian cancer cell line and in the human ovarian carcinoma cell lines, A2780, COV362 and SKOV3. In addition, the same technique was used to determine expression of other TLRs in ID8-VegfA cells, and chemokine receptors in isolated DCs. Reactions were set up in a 25μl total volume, with each one using PCR Reaction Buffer, Mg^+2^, dNTPs, and *Taq* DNA Polymerase (all from Invitrogen; Grand Island, NY). The primers were designed using PrimerBLAST (NCBI) and were used at a concentration of 0.4 μM. For analysis, each reaction product was mixed with 10μl of 6x loading buffer (Invitrogen; Grand Island, NY), and resolved on a 1.5% agarose gel, run at 100V for thirty minutes in 1xTAE buffer, using the 100 bp DNA Ladder (Invitrogen; Grand Island, NY) for reference. The primer sequences for each gene of interest, together with the amplicon size are listed in Table 1.

**Table 1:** Sequence, replicon size and gene bank access number for the primers used in qualitative and quantitative PCR analysis

Target Gene	Primer Sequence	Replicon Size (Bp)	Gene Bank
Mouse(m) CCL3/MIP-1α	Forward 5′-TTC CAC GCC AAT TCA TCG TT-3′ Reverse 5′-TGG ACC CAG GTC TCT TTG GA-3′	120	NM_011337.2
mCCL4/MIP-1β	Forward 5′-GGC TGC CTT CTG TGC TCC AG-3′ Reverse 5′-GCT GCC GGG AGG TGT AAG AGA-3′	85	XM_011248832.1
mCCL5/RANTES	Forward 5′-CTC ACT GCA GCC GCC CTC T-3′ Reverse 5′-GGC ACG AGG CAG CTC TAG G-3′	102	M77747.1
mCCL19/ELC/MIP-3β	Forward 5′-TCG TGA AAG CCT CCG CTA CCT-3′ Reverse 5′-GGA GGT GCA CAG AGC TGA TAG GCC-3′	97	NM_011888.2
mCCL21/SLC/Exodus-2	Forward 5′-CCA TCC CGG CAA TCC TGT TC-3′ Reverse 5′-TCT GCA CCC AGC CTT CCT CA-3′	81	NM_011335.2
mCXCL12/SDF1α	Forward 5′-TGT GCC CTT CAG ATT GTT GC-3′ Reverse 5′-TCC TTT GGG CTG TTG TGC TT-3′	125	NM_021704.3
mCCR4	Forward 5′-GCC ATC GTG CAC GCG GTA TT-3′ Reverse 5′-GCC TGG GAG GGA GGC AAA CA-3′	105	XM_006511932.3
mCCR5	Forward 5′-GAA GAG GCA CAG GGC TGT GAG G-3′ Reverse 5′-TGG AAG GTG GTC AGG AGG AGG A-3′	98	NM_009917.5
mCCR6	Forward 5′-TGG GTC TTT CGG ACT TGG TTC G-3′ Reverse 5′-TGG GCA GTT CAG CCA CACT CTCA-3′	95	AK154413.1
mCCR7	Forward 5′-GCC ATC GTC CAG GCC GTG T-3′ Reverse 5′-GCA GCTC CGG GAT GGA GAG G-3′'	115	NM_001301713.1
mCX3CR1	Forward 5′-GGC CAC CTT GCC CTT CTG GA-3′ Reverse 5′-TGC CCC CAA AGA AGC CAA TGA-3′	107	XM_011242934.1
mCXCR4	Forward 5′-CCT GGC CTT CAT CAG CCT GGA-3′ Reverse 5′-CTG GGA TCC AGA CGC CCA CA-3	113	XM_006529113.3
mGAPDH	Forward 5′-CCT GCA CCA CCA ACT GCT TA-3′ Reverse 5′-CAT GAG TCC TTC CAC GAT ACC A-3′	74	GU214026.1
hGAPDH	Forward 5′-CCA CCA TGG AGA AGG CTG GGG CTC-3′ Reverse 5′-GGG GCA TCA GCA GAG GGG GCA-3′	58	NM_001289746.1
mIL-6	Forward 5′-CCG CTA TGA AGT TCC TCT CTG CAA-3′ Reverse 5′-TGA AGT AGG GAA GGC CGT GGT-3′	88	NM_031168.2
mMDA-5	Forward 5′-TCC AGA CGA TGA CGG TGT GCA G-3′ Reverse 5′-CTG CCT CCT TGT TGG TGT GAT GG-3′	77	XM_006500187.3
Human*(h)* MDA-5	Forward 5′-TTG GAC TCG GGA ATT CGT GG-3′ Reverse 5′-ATG CCC CAG ACC TCC TTC TC-3′	498	NM_022168.3
hMDA5	Forward 5′-TTC AGG CAC CAT GGG AAG TG-3′ Reverse 5′-TGG CTG GGC AAC TTC CAT TT-3′	105	NM_022168.3
mPKR	Forward 5′-TTC GGG ACC TCC ACA TGA CA-3′ Reverse 5′-CGT TTC TTG CCT CCT GCT TTG-3′	81	XM_006523863.3
hPKR	Forward 5′-AAG CAA AAC AAT TGG CCG CT-3′ Reverse 5′-TTT ACT TCA CGC TCC GCC TT-3′	475	XM_011532987.2
hPKR	Forward 5′-GGG GAA AAC GAA ACT GAG AAC C-3′ Reverse 5′-CCG CAA GTC ACA AAG TAT GAG C-3′	106	NM_002759.3
mRIG-1	Forward 5′-CAT GGC TTC CTC CGC GGT CT-3′ Reverse 5′-CAC GGG ACC CAC TGC CTC AG-3′	79	AY553221.1
hRIG-I	Forward 5′-AGT CCT GAG CAA CAG TGA G-3′ Reverse 5′-TAC TTT CAG CGA GAG AGG-3′	489	NM_004585.4
mTLR2	Forward 5′-CAT CGC TTT TTC CCA ATC TCA CAA-3′ Reverse 5′-CAT TGA GAG AAG TCA GCC CAG CA-3′	108	XM_006501460.3
mTLR3	Forward 5′-AAG CAA CCC TTT CAA AAA CCA GAA GA-3′ Reverse 5′-CTC CAG TTG GAC CCC CGT TC-3′	97	XM_006509278.3
mTLR3	Forward 5′-GAA TCA CAA TCG CGC ACC AA-3′ Reverse 5′-TCA GGT TCG TGC AGA AGA CAA-3′	499	XM_006509278.3
hTLR3	Forward 5′-AGT GCC GTC TAT TTG CCA CA-3′ Reverse 5′-TCG TGC AGA AGG CAA AGG TT-3′	461	NM_003265.2
hTLR3	Forward 5′-GGA AAG GCT AGC AGT CAT CC-3′ Reverse 5′-GTG GTG GAG GAT GCA CAC A-3′	105	NM_003265.2
mTLR4	Forward 5′-GGA ATG TCA TCA GGG ACT TTG CTG-3′ Reverse 5′-CCT GAC ACC GGG AAG CTT GAA-3′	98	BC029856.1
mTLR7	Forward 5′-TGG GTT TTG CAG GAG CTG GT-3′ Reverse 5′-TGG CTG TCC TGG TAG CCA GTC T-3′	95	XM_011247786.1
hIL6	Forward 5′-CCC TCG AGC CCA CCG GGA ACG-3′ Reverse 5′-GCA ACT GGA CCG AAG GCG CTT GT-3′	94	XM_011515390.2
hIL8	Forward 5′-AAG CTG GCC GTG GCT CTC TTG-3′ Reverse 5′-GTT CTT TAG CAC TCC TTG GCA AAA CTG-3′	86	NM_000584.3
hCCL4/MIP-1β	Forward 5′-GCA CCA ATG GGC TCA GAC CCT CCC-3′ Reverse 5′-GGC TGG GAG CAG AGG CTG CTG G-3′	112	NM_002984.3
hCCL7/MCP-3	Forward 5′-GAA GGA CCA CCA GTA GCC ACT GTC C-3′ Reverse 5′-CCC ACT TCT GTG TGG GGT CAG C-3′	93	NM_006273.3
hRANTES/CCL5	Forward 5′-CCA GTG GCA AGT GCT CCA ACC-3′ Reverse 5′-TCC CGA ACC CAT TTC TTC TCT GG-3′	91	NM_002985.2
hMCSF	Forward 5′-CAC TGC TGC TGA GAT GAA TGA AAC A-3′ Reverse 5′-CTC CAG GCG GGT CTG TAG GC-3′	85	NM_000758.3

### Real time quantitative PCR (qPCR)

qPCR experiments were carried out to determine the knock-down efficiency of the dsRNA receptors (TLR3, MDA5, RIG1, PKR), and also for relative quantification of cytokine and chemokine expression at the RNA level after dsRNA/lipofectamine treatment. In addition, the expression of several cytokines and chemokines in tumor samples, as well as transcripts of chemokine receptors in DCs, were also determined by qPCR. All qPCR experiments were carried out using the MyIQ Thermocycler (BioRad; Hercules, CA) and SYBR Green qPCR Master Mix from Quantas Biosciences (Gaithersburg, MD) or with the ThermoScientific Luminaris Green qPCR Mix. GAPDH was used as the reference (housekeeping) gene for all (mouse and human cell) experiments, and RNA levels were normalized using the delta-delta Ct method, obtaining relative RNA quantification values for most experiments. In the case of solid tumor analysis of cytokines and chemokines, we used the absolute quantification method by generating standard curves for our genes of interest and housekeeping genes.

### ELISA analysis of cytokines and chemokines

Cytokine and chemokine expression at the level of protein was determined by antigen capture ELISA studies performed on supernatants of cultured cells. To accomplish this, we used specific anti-mouse capture antibodies: IL-6 (MP5-20F3, eBioscience, San Diego, CA); RANTES/CCL5 (MAB4781), MIP-2 (MAB452), (both R&D Systems, Minneapolis, MN). For detection we used: anti-mouse IL-6 (MP5-32C11, eBioscience, San Diego, CA); RANTES (BAF478), and MIP-2 (BAF452) (all R&D Systems, Minneapolis, MN). For human studies, we used purified (MAB4781) and biotinylated (BAF478) anti-RANTES antibodies respectively (both R&D systems, Minneapolis, MN). In order to quantify cytokine or chemokine levels in culture supernatants, recombinant molecules were used to generate standard curves: murine RANTES (250-07), IL-6 (216-16), and MIP-2 (250-15), and human CCL5/RANTES (300-06) (all purchased from PeproTech, Rocky Hills, NJ). Additional cytokines and chemokines, in both mouse and human samples were analyzed using PeproTech ELISA development kits for the appropriate factor, following the manufacturer's instructions.

### Antibody arrays

The membrane (Mouse or Human Inflammatory Cytokine Microarray, RayBiotech; Norcross, GA) was blocked in 5% non-fat dry milk (NFDM) in PBS-Tween 0.1% (mouse array) or in the provided buffer (human array) for 30 minutes at RT with agitation. A pool of cell supernatants (1 ml) collected after over-night (O/N) dsRNA transfection or mock (lipofectamine) treatments was applied to the membrane and incubated O/N at 4°C with gentle agitation. The samples were decanted and the membrane was washed in accordance to the manufacturer's instructions with the provided wash buffers (RayBiotech, Norcross, GA). The biotin-conjugated antibody cocktail (RayBiotech, Norcross, GA) diluted according to the manufacturer's instructions in 1 ml of block buffer (5% NFDM in PBS-Tween 0.1%), was applied to the membrane and incubated O/N at 4°C with gentle agitation. The antibody solution was then decanted and the membrane was washed according to the manufacturer's instructions. A 1:1000 dilution of streptavidin-HRP (Raybiotech, Norcross, GA) in block buffer was then applied to the membrane and incubated with gentle agitation O/N at 4°C. Following additional washes, ECL detection substrate (Pierce; Rockford, IL) was utilized for chemiluminescent detection, as per the manufacturer's instructions using the ChemiDoc XRS+ system (BioRad, Hercules, CA).

### Immunohistochemistry analysis

Solid tumor samples were snap-frozen in OCT medium (Tissue Tek, Sakura, Torrance, CA) and sections were prepared using a Leica CM1950 Cryostat (Leica Microsystems, Bannockburn, IL). Sections were fixed in cold acetone for 10 minutes, pretreated with 3% H_2_O_2_ for 20 min to block endogenous peroxidase activity and blocked in normal horse serum (Vector Laboratories). Biotinylated goat anti-mouse Exodus-2/CCL21 and goat isotype control (both R&D systems) were used at 1:50 dilution for these studies. Then, the Vectastain ABC kit was applied as described by the manufacturer (Vector Laboratories). Sections were counterstained with Gill's hematoxylin (Vector Laboratories). Images were acquired through a Micropublisher 5.0 Digital CCD Color Camera (Qimaging, Surrey, BC Canada).

### Statistical analysis

For all statistical analyses between differentially-treated groups, GraphPad Instat software was used. Two-tailed Student *t*-tests were employed for comparison between the groups, and ANOVA was performed for all multiple group comparisons, followed by the Tukey-Kramer multiple comparisons test. A p value equal to or less than 0.05 was considered a statistically significant difference. Each group contained data from experiments performed in duplicate or in triplicate as needed to obtain statistically meaningful results.

## SUPPLEMENTARY MATERIALS FIGURES



## Abbreviations

CTL: Cytotoxic T lymphocytes
DCs: Dendritic cells
dsRNA: Double-stranded RNA
ECM: Extracellular matrix components
GM-CSF: Granulocyte-macrophage colony-stimulating factor
MDA5: Melanoma-differentiation associated protein 5
NFDM: Non-fat dry milk
NK: Natural Killer
PAMPs: Pathogen-associated molecular patterns
PKR: Protein kinase R
poly(A:U): Polyadenylic:polyuridylic acid
poly(I:C): Polyinositic:polycytidylic acid
PRR: Pathogen recognition receptor
qPCR: Real time quantitative PCR
RIG1: Retinoic acid-inducible gene 1
S.C.: Subcutaneous
siRNA: Small inhibitor RNA
TADCs: Tumor-associated DCs
TAMs: Tumor-associated macrophages
TLR3: Toll-like receptor 3
TME: Tumor microenvironment
