# Supplementary material for: Modulation of double-stranded RNA pattern recognition receptor signaling in ovarian cancer cells promotes inflammatory queues
Source: Oncotarget. 2018 Nov 30;9(94):36666–83. doi: 10.18632/oncotarget.26378 (PMC6291178; doi:10.18632/oncotarget.26378)
Supplement: Supplementary file 1 [file oncotarget-09-36666-s001.pdf]

Modulation of double-stranded RNA pattern recognition receptor signaling in ovarian cancer cells promotes inflammatory queues

SUPPLEMENTARY MATERIALS

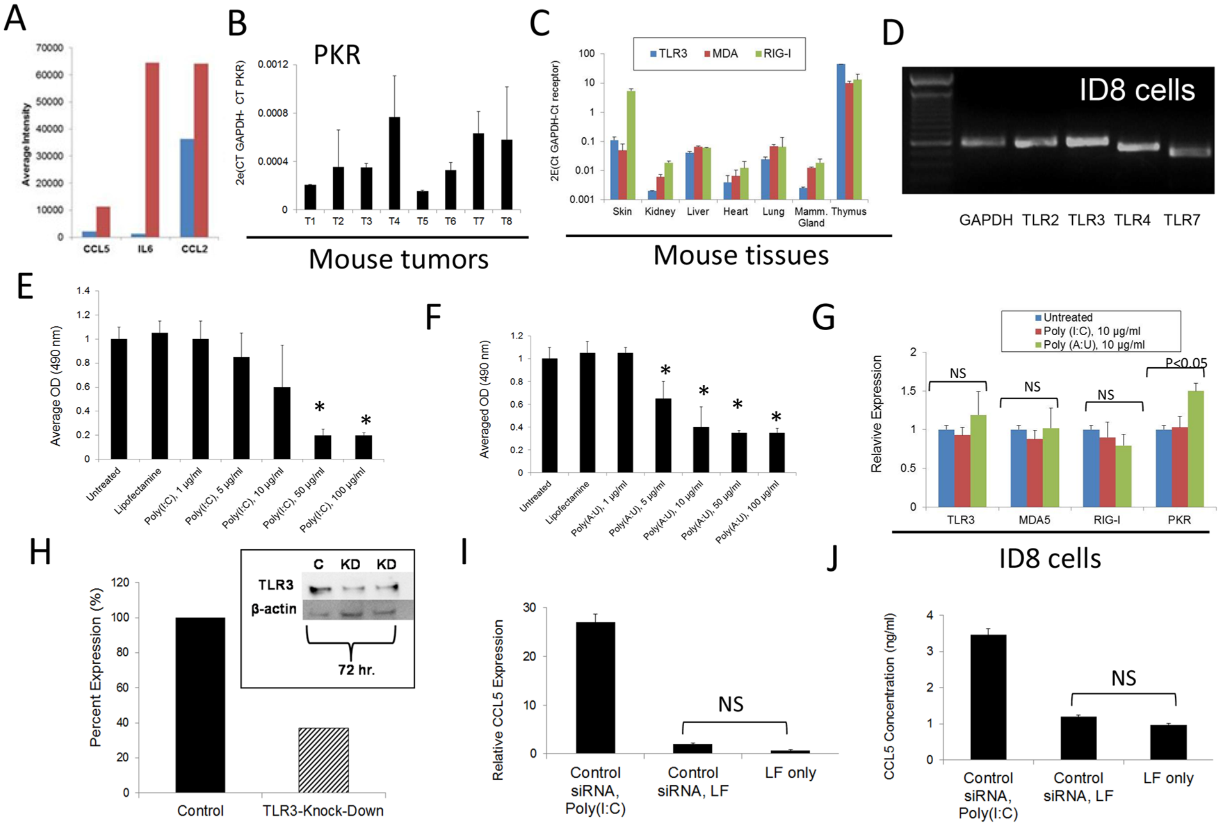

Supplementary Figure 1: (A) Densitometry analysis of antibody arrays performed on supernatants of cancer cells treated with poly(I:C) (red bars) or controls (blue bars). Densitometry analysis was performed by using the ImageJ software and data represented as bars for comparison. **(B)** Expression of PKR at the level of RNA in eight individual mouse ID8-VegfA ovarian cancer solid tumors (T1-8) as determined by qPCR analysis. **(C)** Expression of dsRNA PRRs in several organs of healthy mice as determined by qPCR analysis. **(D)** Qualitative PCR analysis of several TLRs expressed by the mouse ovarian cancer cell line ID8-VegfA. **(E** and **F)** Cell viability (MTS) analysis of ID8 ovarian cancer cells subjected to different doses of poly (I:C) (E) or poly (A:U) (F). The results depicted here are representative of three independent experiments. **(G)** qPCR analysis of dsRNA receptors levels in ID8 cells upon stimulation with poly (I:C) or poly (A:U). Error bars represent +/- SE. The results depicted here are representative of three independent experiments. **(H)** Protein levels of TLR3 in ID8-VegfA ovarian cancer cells upon siRNA inhibition. Box shows the results of a western blot gel. Bars represent quantification analysis of western blot gels. The results depicted here are representative of three independent experiments. **(I** and **J)** Expression of RANTES/CCL5 at the RNA (I) or protein (J) levels by ID8 cells upon transfection with control siRNA or lipofectamine alone. Control siRNA transfection does not activate RANTES/CCL5 expression. The results depicted here are representative of three independent experiments.

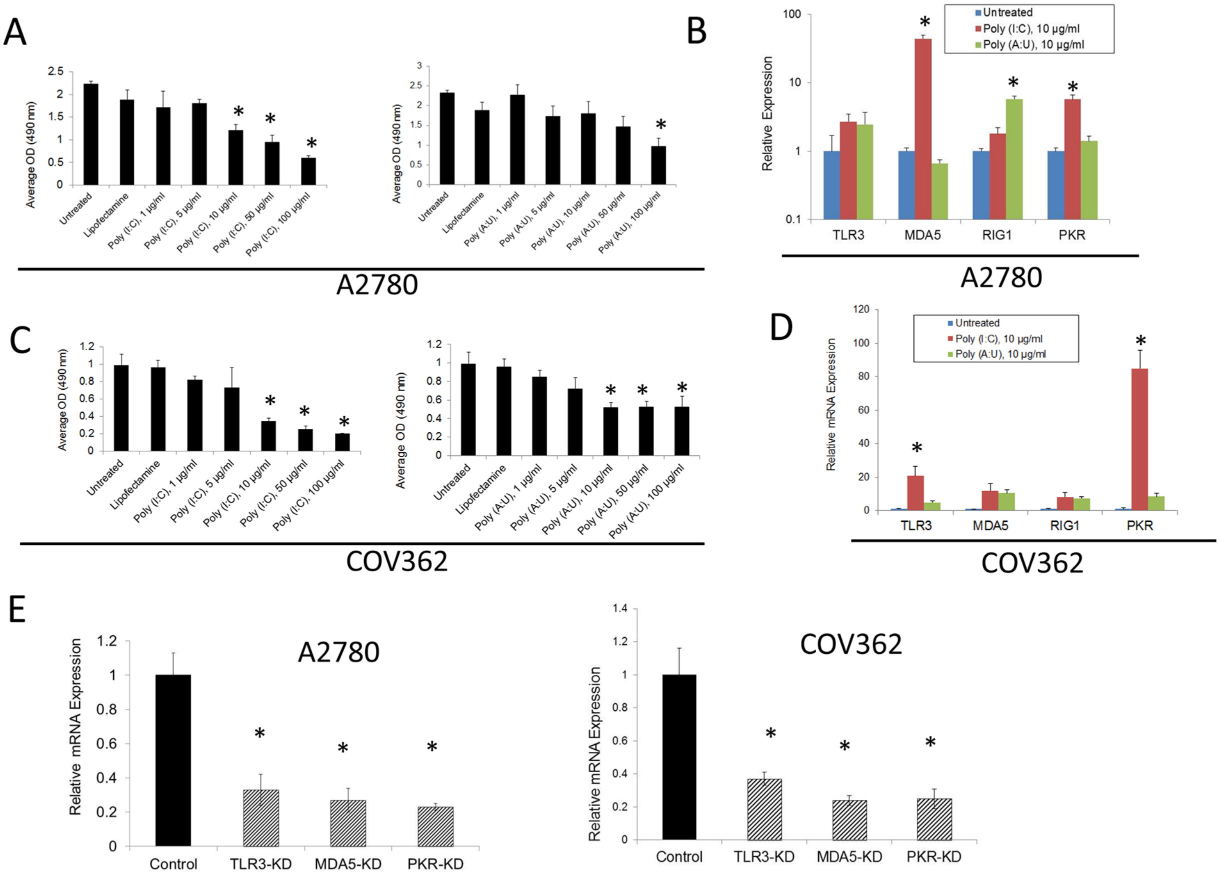

Supplementary Figure 2: (A) Cell viability (MTS) analysis of A2780 ovarian cancer cells subjected to different doses of poly (I:C) or poly (A:U). The results depicted here are representative of three independent experiments. **(B)** qPCR analysis of dsRNA receptors levels in A2780 cells upon stimulation with poly (I:C) or poly (A:U). Error bars represent +/- SE. The results presented are representative of three independent experiments. **(C)** MTS analysis of COV362 ovarian cancer cells subjected to different doses of poly (I:C) or poly (A:U). The results depicted here are representative of three independent experiments. **(D)** qPCR analysis of dsRNA receptors levels in COV362 cells upon stimulation with poly (I:C) or poly (A:U). Error bars represent +/- SE. The results depicted here are representative of three independent experiments. **(E)** Inhibition of dsRNA receptors at the level of RNA as determined by qPCR upon specific siRNA transfection. Error bars represent +/- SE. The results depicted here are representative of three independent experiments.

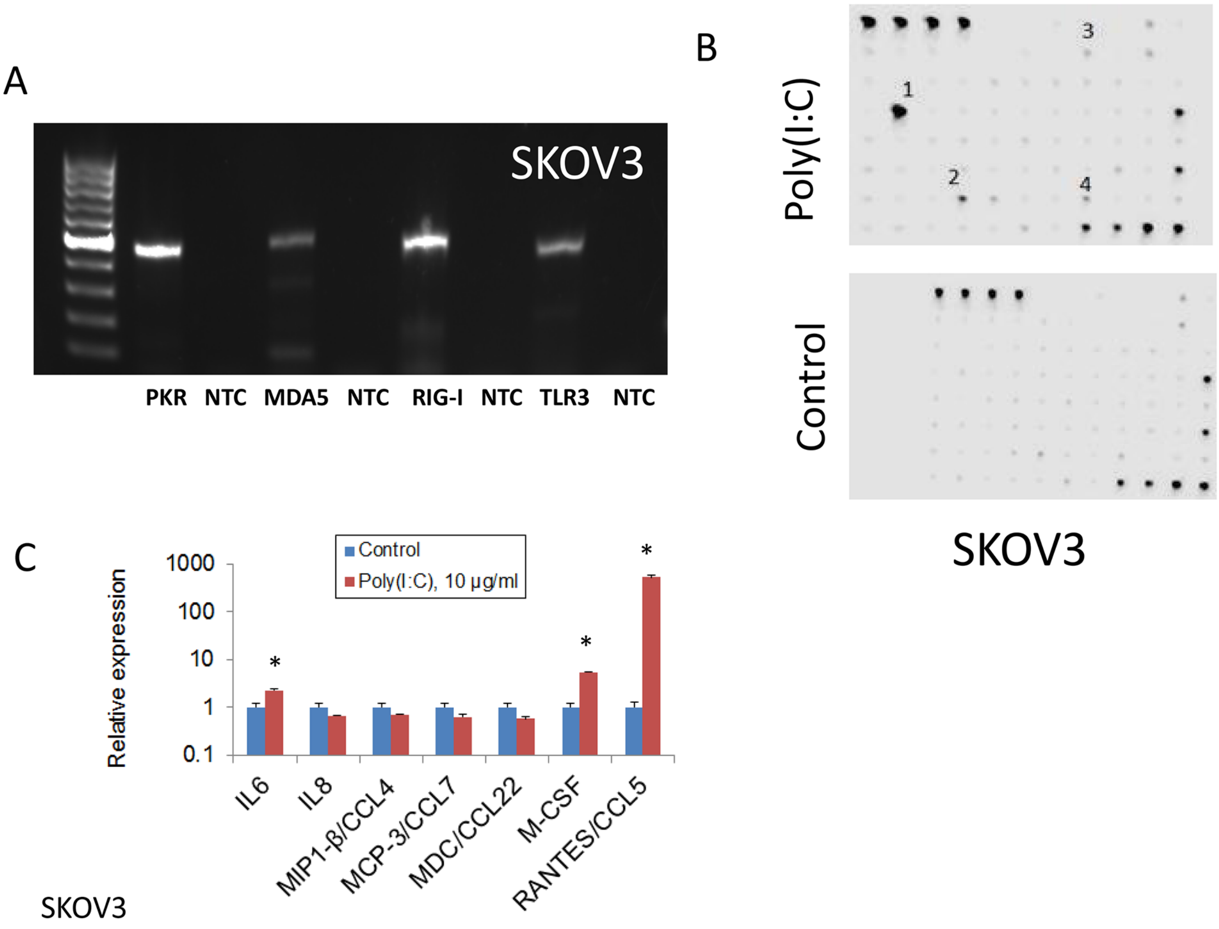

Supplementary Figure 3: (A) RNA was extracted from SKOV3 human ovarian cancer cells and the expression of several dsRNA PRRs analyzed by qualitative PCR. NTC: Non-template control. **(B)** Human SKOV3 ovarian cancer cells were treated for 24 hr. with 10 µg/ml of poly (I:C) admixed with lipofectamine or an equivalent amount of media admixed with lipofectamine as control. Pooled supernatants of two independent experiments were pooled evaluated by antibody arrays. As depicted, a qualitative upregulation in specific chemokines and cytokines was observed. These correspond to 1: RANTES/CCL5; 2: IP-10; 3: IL-6, and 4: MIF, according to the Human Cytokine Array 5 (RayBiotech). **(C)** qPCR analysis of SKOV3 cells subjected to poly (I:C) transfection as above or lipofectamine control. Error bars represent +/- SE. * $p<0.05$.
